# Functional Analysis and Expressional Characterization of Rice Ankyrin Repeat-Containing Protein, *Os*PIANK1, in Basal Defense against *Magnaporthe oryzae* Attack

**DOI:** 10.1371/journal.pone.0059699

**Published:** 2013-03-26

**Authors:** Shaoliang Mou, Zhiqin Liu, Deyi Guan, Ailian Qiu, Yan Lai, Shuilin He

**Affiliations:** 1 College of Life Science, Fujian Agriculture and Forestry University, Fuzhou, Fujian, China; 2 National Education Minster Key Laboratory of Plant Genetic Improvement and Comprehensive Utilization, Fujian Agriculture and Forestry University, Fuzhou, Fujian, China; China Agricultural University, China

## Abstract

The ankyrin repeat-containing protein gene *OsPIANK1* (AK068021) in rice (*Oryza sativa* L.) was previously shown to be upregulated following infection with the rice leaf blight pathogen *Xanthomonas oryzae* pv *oryzae* (*Xoo*). In this study, we further characterized the role of *OsPIANK1* in basal defense against *Magnaporthe oryzae* (*M.oryzae*) by 5′ deletion analysis of its promoter and overexpression of the gene. The promoter of *OsPIANK1* with 1,985 bps in length was sufficient to induce the *OsPIANK1* response to inoculation with *M.oryzae* and to exogenous application of methyl jasmonate (MeJA) or salicylic acid (SA), but not to exogenous application of abscisic acid (ABA). A TCA-element present in the region between −563 bp and −249 bp may be responsible for the *OsPIANK1* response to both *M.oryzae* infection and exogenous SA application. The JERE box, CGTCA-box, and two MYB binding sites locating in the region between −1985 bp and −907 bp may be responsible for the response of *OsPIANK1* to exogenous MeJA. *OsPIANK1* expression was upregulated after inoculation with *M.oryzae* and after treatment with exogenous SA and MeJA. Overexpression of *OsPIANK1* enhanced resistance of rice to *M.oryzae*, although it did not confer complete resistance. The enhanced resistance to *M.oryzae* was accompanied by enhanced transcriptional expression of SA- and JA-dependent genes such as *NH1, WKRY13, PAL, AOS2, PR1b,* and *PR5*. This evidence suggests that *OsPIANK1* acted as a positive regulator in rice basal defense mediated by SA- and JA-signaling pathways.

## Introduction

Plants naturally co-exist with numerous and varied microbial pathogens and the diseases that can result represent a heavy loss in crop productivity worldwide. In consequence, plants have evolved effective immune systems to defend themselves against the attack of microbial pathogens. Two interconnected modes of the innate immune system are used by plants to defend against phytopathogens. The first mode, pattern-triggered immunity (PTI), results from the activation of plant cell-surface pattern recognition receptors (PRRs) following the exposure of these receptors to molecular patterns common to many types of microbes (pathogen-associated molecular patterns [PAMPs]). Infection by a particular pathogen can be blocked by PTI. Pathogens have evolved effector molecules that are secreted into plant cells and suppress PTI, and, in turn, plants have evolved resistance (R) proteins that recognize the effectors and activate the second mode of the innate immune system, effector-triggered immunity (ETI), in a gene-to-gene way [1]–[4]. In addition to the protein-protein interactions mediated by PRRs, R proteins, PAMPs, and effectors, other proteins such as the leucine-rich repeat (LRR) domain-containing proteins [5]–[7] and the ankyrin (ANK) domain-containing proteins [8], [9] are important in plant defense.

The ANK domain is one of the most common protein motifs in eukaryotic proteins. Repeated ankyrin domains are ubiquitous, can function in protein-protein interactions, and may act as molecular chaperones for a class of membrane-bound proteins in plants [10], [11]. In addition to the ANK-repeat domain, ANK proteins often contain several other functional domains, for examples, PEST, calmodulin binding motifs, and Ring Finger domain [12], [13]. Mediation of protein-protein interactions by the ANK domain is involved in a number of physiological and developmental responses such as the cell cycle, cell differentiation [14]–[19], plastid differentiation [14], pollen germination and pollen tube growth [20], lateral root development [21], leaf morphogenesis [16], targeting of proteins to the plastid outer envelope [22], [23], grana formation [24], anthocyanin biosynthesis [25], nitrogen-fixing symbiosis in root nodules of *Lotus japonicus* [26], plant microbe interaction [27], [28], male-female gamete recognition [29], plant immunity [8], [9], [30]–[36], plant resistance to abiotic stress associated with Cd [37]–[39], regulation of a plant potassium channel [40], and ethylene signaling [41], [42]. In higher plants, ANK proteins constitute a large multi-gene family. For example, 175 and 105 ANK repeat genes have been found in rice and the *Arabidopsis* genome, respectively [12], [13]. However, the function and the underlying mechanism of most of the genes of the ANK family of proteins remains poorly understood.

Here, we report on a study of the ANK domain in the plant immune response, using rice as a model system. Rice is one of the most agriculturally important crops worldwide, but approximately a million tons are lost to disease each year. Better understanding of the genetic mechanisms of rice defense responses could lead to better agricultural management and reduced losses due to diseases.

The *Os*ANK proteins in rice can be classified into 10 subfamilies. Expression profile analysis of *Os*ANK proteins suggested that members of this gene family may play important roles in pollination, fertilization, and signaling [12]. Among all of the 175 known ANK-repeat genes in rice, only a few members have been functionally characterized. The ANK-repeat domain-containing XA21 binding protein 3 was required for full *Xa21*-mediated disease resistance [33], *Os*CBT, containing three ANK repeats, is a transcription activator modulated by CaM [43], *Os*BIANK1, encoding a plasma membrane-anchored ANK-repeat protein, was induced by treatment with either benzothiadiazole or by infection with *Magnaporthe grisea* [31]. *Os*PIANK1, an ankyrin protein in rice, was late up-regulated by infection of the rice leaf blight pathogen *Xoo* at both early and late infection stages, and had different expression patterns against the attack of *Xoo* [44]. Both PTI and ETI are accompanied by major transcriptional reprogramming, which is considered a key step of the plant defense program [45], [46]. Up to 25% of all *Arabidopsis* genes respond to pathogen infection by altering their transcript levels [47], [48]. Genetic evidence indicates that genes that are transcriptionally up-regulated during plant immune responses are important in disease resistance [49]–[51]. This reasoning suggests that *OsPIANK1* plays an important role in rice immunity, although there is no direct evidence for this role. In the present study, we performed functional identification of *OsPIANK1* by its overexpression in rice and expressional characterization by a 5′ deletion assay of its promoter. The results suggest that *OsPIANK1* is a positive regulator in the rice basal defense mediated by the SA and JA signaling pathways.

## Materials and Methods

### Isolation of the Full Length cDNA and Promoter of *OsPIANK1*

The full length cDNA of *OsPIANK1* was amplified by reverse transcription-polymerase chain reaction (RT-PCR). Total RNA from leaves of Nipponbare (*Oryza sativa* L. japonica) was extracted using the TRIzol Reagent (Invitrogen, Carlsbad, CA, USA). Moloney Murine Leukemia Virus (M-MLV) Reverse Transcriptase (Invitrogen) was used for cDNA synthesis according to the manufacturer’s protocol. The newly synthesized cDNA was used as the template for PCR amplification with the *OsPIANK1*-specific forward primer (5′-TACGGCATgCATACTCCATCA-3′) and reverse primer (5′-TACATGCATGCAAGCTGTCA-3′). Genomic DNA of Nipponbare was extracted from leaf tissues using the hexadecyltrimethylammonium bromide (CTAB) method. The *OsPIANK1* promoter region was amplified by PCR using the specific primer pair (5′- TTTGCACCTTTTACCCTg-3) and (5′-CGCTTATTAGAGATAAAGAGGA-3′). All of the PCR products was cloned into pMD18-T vector and sequenced. The *OsPIANK1* promoter sequences were analyzed by the PLACE Web Signal Scan program (http://bioinformatics.psb.ugent.be/webtools/plantcare/html/).

### Vectors Construction and Rice Transformation

To construct the overexpression vector of *OsPIANK1*, the open reading frame of *OsPIANK1* was amplified by PCR with *Kpn* ^−^-*Spel* ^−^ (indicated by lowercase letters) linker primers (5′-GGggatccATGCATACTCCATCAAT-3′, 5′- GGactagtTCAGGTCGTCATCACAT-3′) using pMD18T-*OsPIANK1* as the template, which was further cloned into the *Kpn* I and *Spel* I sites of the modified binary expression vector pCAMBIA1390 under the control of the maize ubiquitin promoter.

Serially 5′-deleted *OsPIANK1* promoters were created by PCR using the full-length promoter fragment as templates with four forward primers (-1101∶5′-TATCTGGTACCTTCAGAG-3′, -907∶5′-TTCGGCCAATAGGATGTA-3′, -563∶5′-TGTTGGTCTAATCTTCGG-3′, -249∶5′-TTGCAATATTGACAGCAG-3′) and the reverse primer (5′-CGCTTATTAGAGATAAAGAGGA-3′). The four promoter deletion-GUS fusion constructs were further cloned into the binary destination vector pMDC163 [52] using the Gateway cloning technique.

After verification by sequencing, all of the vectors were transferred into *Agrobacterium tumefaciens* strain EHA105, and then were transformed into Nipponbare rice. The *Agrobacterium*-mediated transformation was performed according to the method of Toki S [53] using vigorously growing callus derived from mature embryos. More than 10 independent transgenic lines were obtained for each vector.

### Pathogen and Exogenous Phytohormones Treatment

To analyze the response of *OsPIANK1* promoter to inoculation with the pathogen and exogenous application of phytohormones, T_1_ transgenic rice plants with the *GUS* reporter gene controlled by the *OsPIANK1* promoter were developed. These plants may have had a single T-DNA insertion since the T1 progeny from individual positive clones exhibited a nearly 3∶1 ratio in resistance to hygromycin. The sterilized T1 transgenic and wild-type rice seeds were germinated for 2 weeks on Murashige and Skoog agar medium with or without 50 mg L ^−1^ hygromycin, respectively. The resulting seedlings were transferred to soil in pots and grown in a greenhouse (25°C/27°C, solar radiation). Rice seedlings were treated at the four-leaf stage. For inoculation with the pathogen, leaves were sprayed with a suspension of conidia (1×10^5^ mL^−1^) of *M.oryzae* strain guy11. For exogenous phytohormone treatment, 100 µM ABA in sterile double-distilled H_2_O, SA (100 µM in 10% ethanol), or 100 µM MeJA (in 10% ethanol) was sprayed on the leaves and the plants were kept in a greenhouse (25°C/27°C, solar radiation) until the samples were harvested at the designated times.

### GUS Activity Assay

Histochemical staining for beta-glucouronidase (GUS) activity was performed as reported previously [54]. Leaves of *OsPIANK1*pro-*GUS* transgenic plants were detached and incubated in GUS reaction buffer (0.2 M phosphate pH 7.0 containing 0.1 M K_3_[Fe(CN)_6_], 0.1 M K_4_[Fe(CN)_6_], 1.0 M EDTA-Na_2_, 0.1% 5-bromo-4-chloro-3-indolyl-beta-D-glucuronide [X-Gluc]) at 37°C for 24 h. For better visualization of the stained tissue, leaves were rinsed at room temperature with an ethanol series for at least 1 h to remove chlorophyll.

Total protein was extracted from the leaf tissue of the *OsPIANK1*pro-*GUS* plants. GUS activity was measured using a spectrophotometer to quantify the rate of release of *p*-nitrophenol (*λ = *415 nm) from *p*-nitrophenyl-*β*-D-glucuronide (PNPG) [54]. GUS activity assays were repeated with at least two independent lines, each with three replicates. When similar results were obtained in repeated experiments, only one experiment has been presented.

### Subcellular Localization of *Os*PIANK1

For *Os*PIANK1 localization, the full-length coding sequence of *OsPIANK1* was amplified and fused in frame to the N-terminus of the green fluorescent protein (GFP) gene to generate the CaMV35S: *Os*PIANK1-GFP construct by Gateway-mediated recombination into the vector pMDC83. The CaMV35S::GFP construct was used as the control. Both constructs were further transformed into *Agrobacterium tumefaciens* strain GV3101. The cells of GV3101 harboring CaMV35S::*Os*PIANK1-GFP constructs was co-infiltrated into *N. benthamiana* leaves. One day after agro-infiltration, the leaves were visualized under a fluorescence microscope (Olympus DP72), with an excitation wave length of 488 nm and a 505–530 nm band-pass emission filter. 4′,6-diamidino-2-phenylindole (DAPI) fluorescence was also imaged using an excitation wavelength of 405 nm and a 435–480 nm band-pass emission filter.

### Quantitative Real-time PCR

For quantitative real-time PCR analysis, total RNA from the leaves of the wild-type (Nipponbare) and transgenic plants were extracted. First-strand cDNA was generated by converting 500 ng total RNA using Primescript RT reagent (perfect real time, TaKaRa), and the cDNA was diluted to 100 µL with water. Real-time PCR using Mastercycler ep *realplex* (Eppendorf, Hamburg, Germany) was performed with SYBR® Premix Ex Taq™ II (perfect real time, TaKaRa). Each reaction mix (25 µL) contained 12.5 µL SYBR Premix Ex Taq, 0.5 µL PCR forward/reverse gene specific primers (10 µM), 2.5 µL diluted cDNA, and 9.5 µL water. For each gene, three experimental replicates were obtained using different cDNAs synthesized from three biological replicates. The thermal cycle used was as follows: one cycle of 30 s at 95°C; 40 cycles of 5 s at 95°C, 34 s at 60°C; one cycle of 15 s at 95°C, 1 min at 60°C, 15 s at 95°C, 15 s at 60°C. Rice *actin* gene (X15865) was used for normalization. The gene-specific primer pairs are listed in Table 1. The relative expression levels were determined as described by Livak and Schmittgen [55].

**Table 1:** Primer information for real-time PCR.

Gene name	Forward primer sequence (5′ –3′)	Reverse primer sequence (5′ –3′)
*PIANK1*	gAgATgTgCCCgTCgCTgTAT;	CACTgTCTgTgCTgAATgATgCC
*PR1b*	ggCAACTTCgTCggACAgA;	CCgTggACCTgTTTACATTTTCA
*PR5*	CAACAgCAACTACCAAgTCgTCTT;	CAAggTgTCgTTTTATTCATCAACTTT
*NH1*	CACgCCTAAgCCTCggATTA;	TCAgTgAgCAgCATCCTgACTAg
*WRKY13*	TCAgTggAgAAgCgggTggTg;	gggTggTTgTgCTCgAAggAg
*AOS2*	CAATACgTgTACTggTCgAATgg;	AAggTgTCgTACCggAggAA
*PAL*	AgCACATCTTggAgggAAgCT;	gCgCggATAACCTCAATTTg
*Actin*	TgTATgCCAgTggTCgTACCA;	CCAgCAAggTCgAgACgAA

### Quantification of *M.oryzae* DNA in Rice Leaves

Rice and fungal DNA were extracted from leaves inoculated with *M. oryzae* and quantified by real-time PCR in accordance with the method of Qi and Yang [56] using two specific primer pairs, which were designed based on the 3′ noncoding region of a *MPG1* gene in *M. oryzae* (5′-GGGATGATGGTGGTGGAGGAC-3′; 5′-GCCAGGTGCTTAGGACGAAAC-3′). The data were normalized to the amount of DNA of a rice *actin* gene (AK060893), which was quantified using the forward primer (5′-GAGTATGATGAGTCGGGTCCAG-3′) and reverse primer (5′-ACACCAACAATCCCAAACAGAG-3′).

## Results

### Expression Pattern of *OsPIANK1* in Response to Pathogen Infection and Exogenous Application with Phytohormones

**Figure 1:**
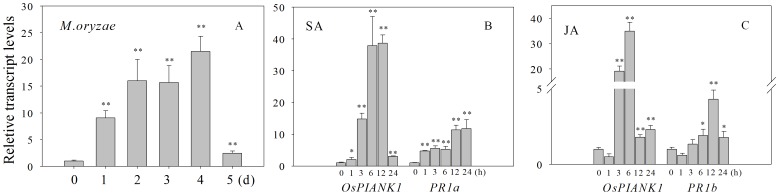
Expression profiles of *OsPIANK1* in response to inoculation with *M.oryzae* or exogenous application of SA and MeJA. Transcript levels of *OsPIANK1* were determined by quantitative real-time PCR. Total RNA was prepared from leaf tissues of 3-week-old seedlings at the times indicated. The expression level of untreated plants at each time was used as the control and assigned a value of 1. Relative expression levels were normalized using the expression of *actin*. Data represents the mean ± SE of three independent biological replicates. (**,* indicate significant differences between the treatment and control at *P*<0.01 or *P*<0.05, respectively, as determined by Student–Newman–Keuls (SNK) test.).

Expression of *OsPIANK1* in rice has been reported to be transcriptionally up-regulated after infection of the rice leaf blight pathogen *Xoo* [44]. We wished to evaluate the possibility that *OsPIANK1* is an important regulator for the rice response to other pathogens and to universal defense signaling molecules such as SA and JA. To this end, the transcriptional levels of *OsPIANK1* following infection with the *M.oryzae* strain guy11 or exogenous application of SA and MeJA were examined by real-time PCR. Transcripts of *OsPIANK1* increased approximately 10-fold 1 day after inoculation with *M. oryzae,* remained high for 2 to 4 days after inoculation, and decreased sharply 5 days after inoculation (Figure 1A). *OsPIANK1* transcripts increased markedly 3–12 h after treatment with SA and declined dramatically 24 h after treatment. SA treatment induced the rice *acidic pathogenesis-related (PR) protein1* (*PR1a*) gene that was used as a comparable control (Figure 1B). In response to MeJA, the mRNA accumulation of *OsPIANK1*was increased remarkably for 3 h, reached a maximal level after 6 h, and decreased sharply 12 h after treatment. MeJA induced the rice *basic PR protein1 (PR1b)* gene that was used as a comparable control (Figure 1C). These results indicate that, in addition to responding to infection by *Xoo*, *OsPIANK1* is involved in the response of rice to *M.oryzae* infection regulated by the signaling pathway mediated by SA and JA.

### Deletion Analysis of the *OsPIANK1* Promoter

**Figure 2:**
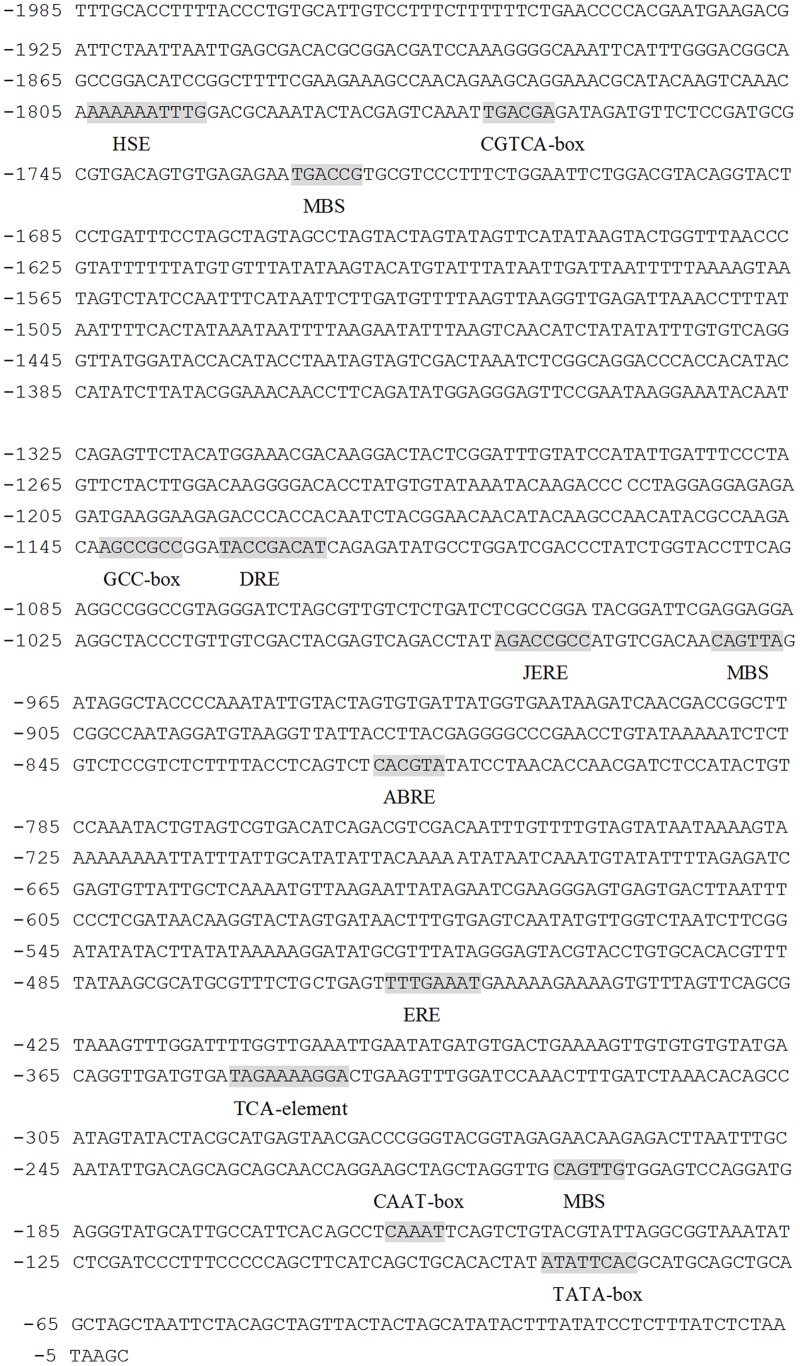
Nucleotide sequence of 5′ flanking promoter regions and stress-related sequence motifs putatively acting as *cis*-elements of the *OsPIANK1* gene. HSE: *cis*-acting element involved in heat stress responsiveness. CGTCA-box: *cis*-acting regulatory element involved in the MeJA-responsiveness. JERE: *cis*-acting regulatory element involved in the MeJA-responsiveness. ERE: ethylene-responsive element. DRE: *cis*-acting element involved in dehydration, low-temp, salt stresses. MBS: MYB binding site involved in drought-inducibility. GCC-box: ethylene-responsive element. ABRE: *cis*-acting element involved in the abscisic acid responsiveness. TCA-element: *cis*-acting element involved in salicylic acid responsiveness.

Gene expression was largely controlled by its promoter. Therefore, deletion analysis of *OsPIANK1* promoter was carried out to dissect the possible molecular mechanism of *OsPIANK1* response to *M.oryzae*, SA, and JA. A 1985-bp upstream region of the *OsPIANK1* open reading frame was isolated from Nipponbare genomic DNA by PCR amplification and *cis*-acting elements in this region were analyzed (http://bioinformatics.psb.ugent.be/webtools/plantcare/html/). Several stress-related sequence motifs putatively acting as *cis*-elements were identified in the *OsPIANK1* promoter (Figure 2). These sequence motifs included two MYB binding sites (MBS) involved in response to drought; a *cis*-acting element involved in response to heat stress (HSE); a *cis*-acting element involved in abscisic acid responsiveness (ABRE); a *cis*-acting element involved in dehydration, low-temperature, and salt stresses (DRE); two ethylene-responsive elements (GCC box and ERE); two *cis*-acting regulatory element involved in the MeJA-responsiveness (JERE and the TCACG motif); and a *cis*-acting element (TCA-element) involved in SA responsiveness.

**Figure 3:**
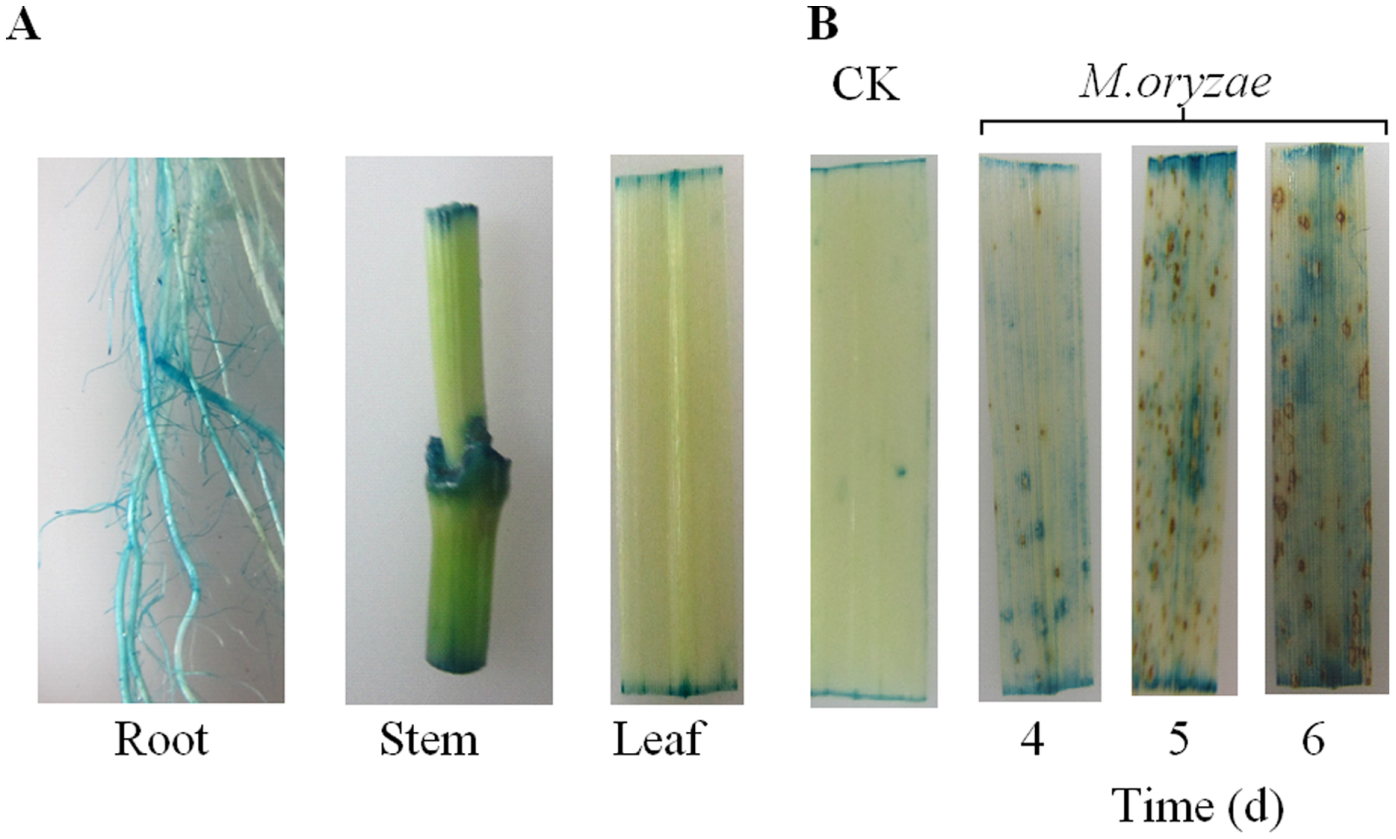
The *OsPIANK1* promoter activities in different organs and its activation in response to *M.oryzae*. (A) Histochemical analysis of GUS activity in different organs of *OsPIANK1pro-GUS* transgenic rice. (B) Histochemical analysis of GUS activity in *OsPIANK1pro-GUS* transgenic rice response to inoculation of *M.oryzae*. The leaves were harvested at 4, 5, and 6 days after inoculation.

To analyze the activity of the *OsPIANK1* promoter in major organs of rice seedlings, the transgenic rice plants of *OsPIANK1pro-β–glucouronidase (GUS)* and their corresponding T_1_ lines were acquired. These lines were phenotypically normal and their growth was similar to wild-type plants. GUS expression was detected histochemically in leaves, stems, and roots of *OsPIANK1pro-GUS* plants (Figure 3A). Next, the activation of the *OsPIANK1* promoter by pathogen infection was assessed. *OsPIANK1pro-GUS* plants were inoculated with *M.oryzae* and the responses of *OsPIANK1* promoter were monitored by determining GUS activity. GUS staining was more intense in *M.oryzae*-inoculated leaves of *OsPIANK1pro-GUS* rice plants 4 to 6 days after inoculation than in the uninoculated leaves (Figure 3B).

**Figure 4:**
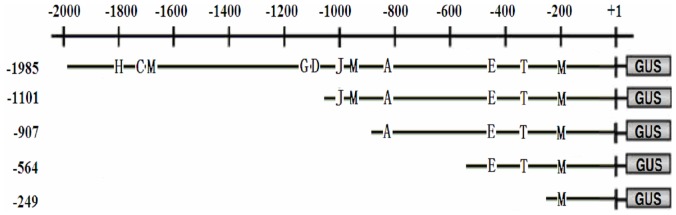
Schematic representation of *OsPIANK1* promoter constructs for assaying GUS (β-glucuronidase) expression in transgenic rice leaves. The serially 5′ -deleted promoter constructs of the *OsPIANK1* gene were fused to the GUS reporter gene in the vector pCMDC163. H: HSE, *cis*-acting element involved in heat stress responsiveness. C: CGTCA-box, *cis*-acting regulatory element involved in the MeJA-responsiveness. J: JERE, *cis*-acting regulatory element involved in the MeJA-responsiveness. E: ERE, ethylene-responsive element. D: DRE, *cis*-acting element involved in dehydration, low-temp, salt stresses. M: MBS, MYB binding site involved in drought-inducibility. G: GCC-box, ethylene- responsive element. A: ABRE, *cis*-acting element involved in the abscisic acid responsiveness. T: TCA-element, *cis*-acting element involved in SA responsiveness.

**Figure 5:**
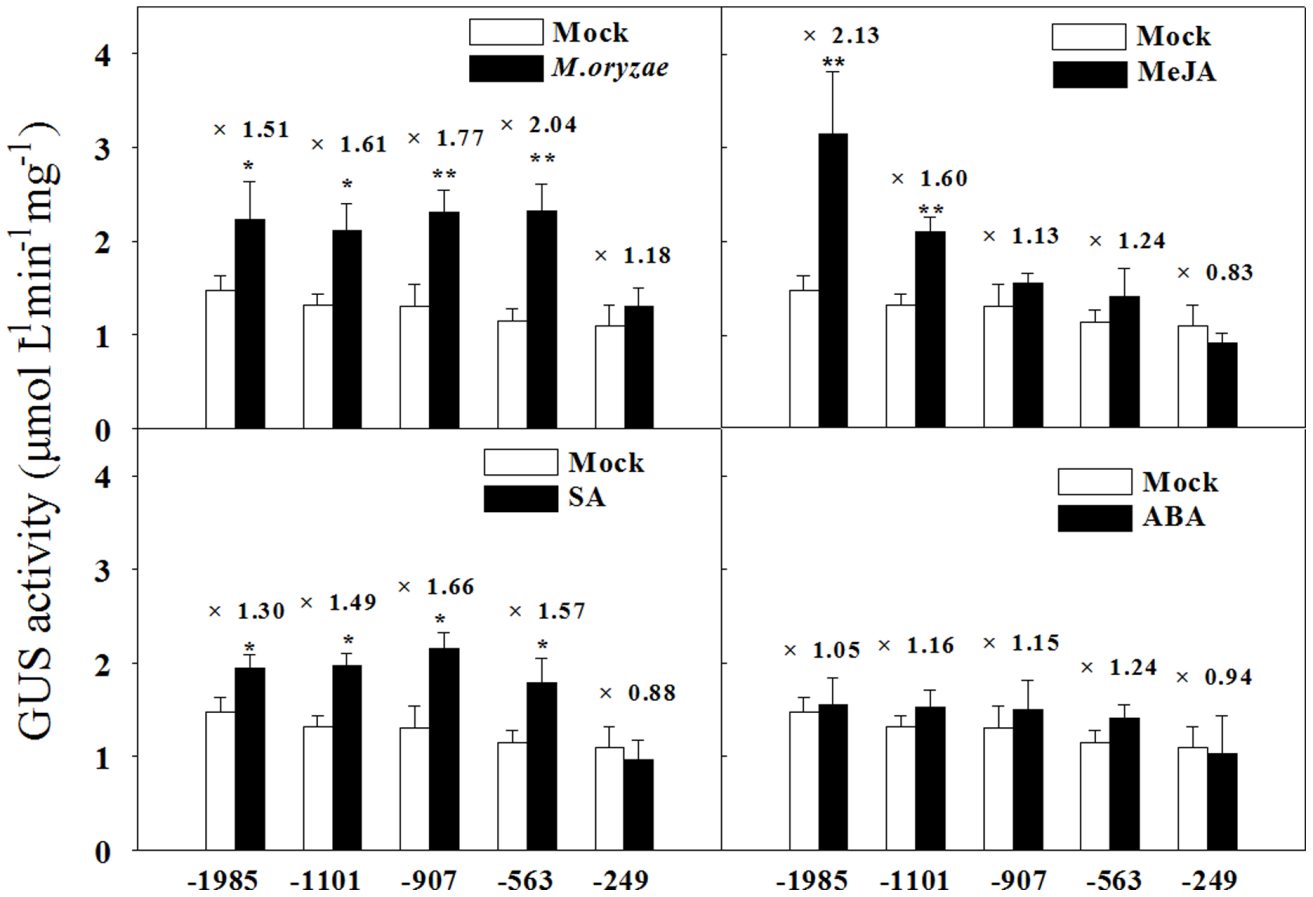
*OsPIANK1* promoter activation in response to inoculation with *M.oryzae* or exogenous application of MeJA, SA or ABA. The leaves were harvested at 6 days after inoculation with *M.oryzae* and 12 h after treatment with the phytohormones. The numbers over the bars indicate the increase in induction of GUS activity compared to the mock. Data represent the means ± SE from the leaf extracts collected from three experimental plant units. (**, *indicate significant differences between the untreated (mock) and treated plants at *P*<0.05 and *P*<0.01, respectively, as determined by the SNK test.).

To perform 5′ deletion analysis of the *OsPIANK1* promoter, serially 5′-deleted *OsPIANK1* promoters were created by PCR (Figure 4), cloned into the vector pMDC163 upstream of the *GUS* reporter gene, and transformed into rice via the *Agrobacterium*-mediated method. The transgenic rice plants and their corresponding T_1_ lines were acquired and detected by PCR. A 5′ deletion assay of the *OsPIANK1* promoter was performed with the transgenic plants. GUS expression in response to infection with *M.oryzae* or exogenous SA was similar for the following promoters: *OsPIANK1pro*-1985 bp, *OsPIANK1pro*-1101 bp, *OsPIANK1pro*-907 bp, and *OsPIANK1pro*-564 bp but different between these and *OsPIANK1pro*-249 bp (Figure 5), suggesting that the *cis*-elements responsible for the response of *OsPIANK1* to *M.oryzae* infection and exogenous SA locate in the region between -564 bp and -249 bp. GUS expression in response to exogenous MeJA differed between the promoters *OsPIANK1pro*-1985 bp and *OsPIANK1pro*-1101 bp, as well as between *OsPIANK1pro*-1101 bp and *OsPIANK1pro*-907 bp (Figure 5). Thus, at least two *cis*-elements may be responsible for the response of *OsPIANK1* to exogenous MeJA. These may locate in the region between −1985 bp and −907 bp.

### A Transiently Expressed *Os*PIANK1-GFP Fusion Protein is Localized in the Nucleus

**Figure 6:**
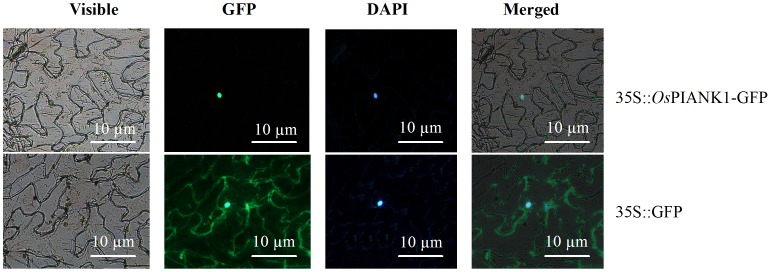
Nuclear localization of *Os*PIANK1 protein in *N. benthamiana* leaves. *Os*PIANK1–GFP exclusively localized in the nucleus of cells in *N. benthamiana* leaves. Only the green fluorescent (GFP) localized throughout the whole cells. Cells were detected for GFP fluorescence by fluorescence microscopy 48 h after agroinfiltration.

The full-length cDNA of *OsPIANK1* was amplified with a specific primer pair of *OsPIANK1* by PCR with cDNA synthesized from the total RNA of rice leaves inoculated with *M. oryzae.* To determine the subcellular localization of *Os*PIANK1, the construct of a Cauliflower mosaic virus 35S-controlled *Os*PIANK1-green fluorescent protein chimaera (CaMV35S::*Os*PIANK1-GFP) and the control vector (CaMV35S::GFP) were constructed, transformed into *Agrobacterium* strain GV3101, and infiltrated into *N. benthamiana* leaves. Localization of the *Os*PIANK1-GFP fusion protein was visualized exclusively in the nuclei (Figure 6).

### *OsPIANK1* Overexpression Enhanced the Resistance of Rice to Infection by *M.oryzae*

**Figure 7:**
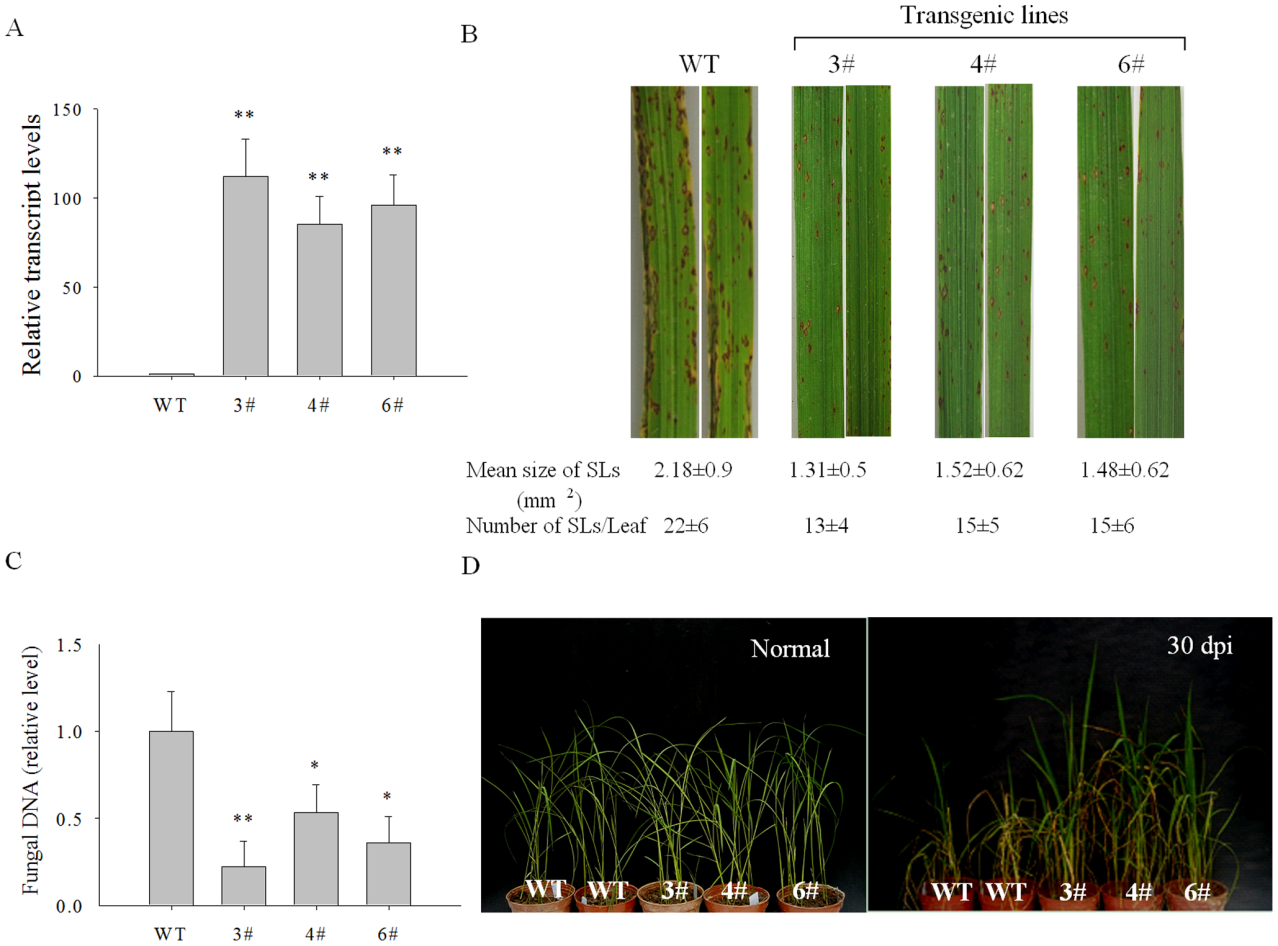
Analysis of resistance to blast fungus in *OsPIANK1*-overexpressing (OE) rice plants. (A) Quantitative PCR analysis of *OsPIANK1* expression in WT and OE plants (3#, 4#, and 6#). Data represent means ± SE of three independent experiments. (B) Lesions in leaves at 6 days after inoculation. The number of expanding lesions (Els) with an area greater than 0.5 mm^2^ per leaf and their mean areas were determined using 10 leaves for WT (Nipponbare) and *OsPIANK1*-OE plants. Values represent means ± SE. (C) The amount of *M. oryzae* DNA in the WT and *OsPIANK1*-OE rice leaves. The leaves were harvested at 6 days after inoculation. Values represent the mean ± SE of three independent experiments. (D) Symptoms of rice blast in *OsPIANK1*-OE and WT rice plants grown in the greenhouse at 30 days after inoculation with spores of *M. oryzae* strain guy11. (**,*indicate significant differences between WT and *OsPIANK1*-OE plants at *P*<0.01 or *P*<0.05, respectively, as determined by the SNK test.).

We wished to characterize the function of *OsPIANK1* in rice against *M. oryzae* attack. First *OsPIANK1*-overexpression (OE) lines were generated. From these, three lines (lines 3#, 4#, and 6#) were chosen based on their high transcript levels of *OsPIANK1.* The function of *OsPIANK1* was studied in T_2_ generations of those transgenic lines compared to WT rice plants. The expression of *OsPIANK1* in the three *OsPIANK1*-OE lines was higher than in WT plants (Figure 7A).

Three-week-old plants of *OsPIANK1*-OE transgenic and WT plants were inoculated with *M.oryzae* strain guy11 and evaluated for symptoms of rice blast 6 days later. The three *OsPIANK1*-OE lines had fewer and smaller expanding lesions (Figure 7B) with less fungus in the leaves (Figure 7C) than WT plants. *OsPIANK1*-OE and wild-type rice plants, both at the four-leaf stage, were inoculated with spores of *M.oryzae* strain guy11 and grown in a greenhouse. The *OsPIANK1*-OE and WT rice plants began to wilt at 15 days after inoculation and some of the WT plants were dead at 30 days after inoculation. In contrast, all of *OsPIANK1*-OE rice plants survived with slight disease symptoms (Figure 7D). These results suggest that *OsPIANK1-*OE enhanced resistance to *M.oryzae*.

### Overexpression of *OsPIANK1* Upregulated the Expression of Defense Marker Genes

**Figure 8:**
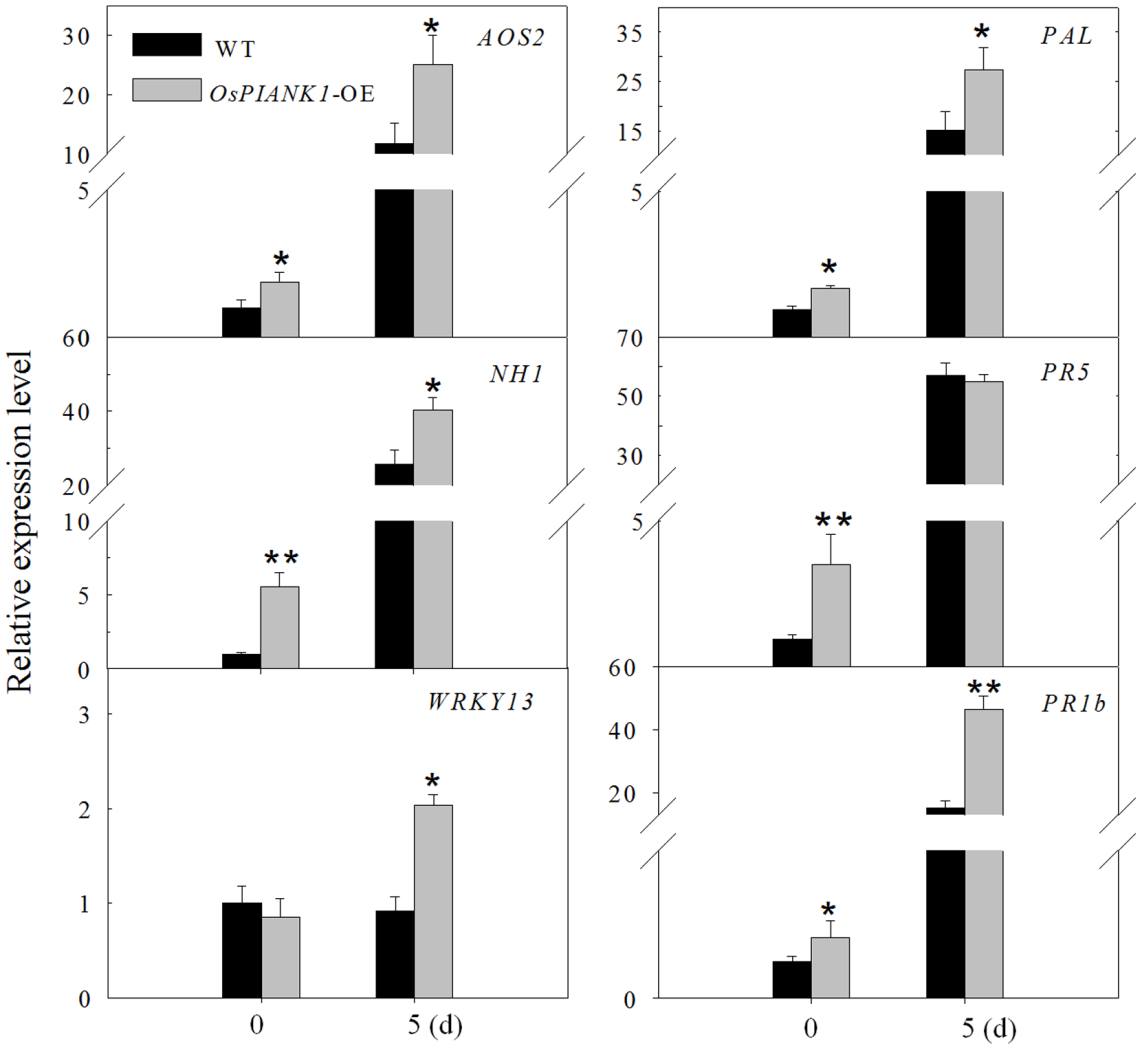
Overexpression of *OsPIANK1* inﬂuenced the expression of a set of genes functioning in SA- and JA-associated pathways during disease resistance against *M.oryzae* strain guy11 analyzed by quantitative real time-PCR. Bars represent mean ± SE of three biological replicates. (**, *indicate significant differences between the *OsPIANK1-*OE and wild-type plants at the same time at *P*<0.01 or *P*<0.05, respectively, as determined by the SNK test.).

The mode of action of *OsPIANK1* in rice was studied by comparing the transcriptional expression of a set of pathogen-induced genes involved in defense in *OsPIANK1*-OE rice seedlings inoculated with *M.oryzae* strain guy11 and in un-inoculated controls. The genes included phenylalanine ammonia lyase (*PAL*; X87946), *Arabidopsis NPR1* homolog 1 (*NH1*; AY9123983), thaumatin-like protein (*PR5*; X68197), basic PR protein 1 *(PR1b*; U89895), *OsWRKY13* (EF143611), and allene oxide synthase 2 (*AOS2*; AY062258). The transcriptional expression of *PAL, NH1, PR1b,* and *AOS2* was enhanced by overexpression of *OsPIANK1* in both inoculated and control seedlings. Expression of PR5 was transcriptionally enhanced only in the control seedlings while *WRKY13* was enhanced only in the inoculated seedlings (Figure 8).

## Discussion

One hundred seventy-five *Os*ANK proteins have been identified in rice, which have been classified into 10 subfamilies based on their domain compositions. Seventy-three (41.7%) belong to subfamily ANK-M, a classification based on the presence of the ANK domain in the absence of other known functional domains. Members of the other subfamilies contain not only the ANK domain but several other known functional domains [12]. The only distinguishing feature of the *Os*PIANK1 protein is its three ANK domains; therefore, it may belong to the ANK-M subfamily. Little is known about the ANK-M rice proteins, and this report may be the first characterization of a protein in this subfamily.

Plant immune responses are accompanied by major transcriptional reprogramming and the genes involved appear to have important roles in the immune response [45]–[50], [57]. *OsPIANK1* was upregulated following infection by the rice leaf blight pathogen *Xoo* [44], making it a likely player in the immune response of rice to pathogens. In the present study, *OsPIANK1* also responded to *M. oryzae*, exogenous SA, and exogenous MeJA, suggesting that *OsPIANK1* is a regulator in basal defense or PTI of rice against different pathogens. Transcriptional expression of genes is largely regulated by *cis*-elements in their promoters. Some stress-related *cis*-elements: the CGTCA-box and JERE, involved in the MeJA-responsiveness [58], [59]; the ethylene-responsive element [60]; the GCC-box [61]; the TCA-element, involved in SA responsiveness [62]; MBS, involved in drought responsiveness [63]; and HSE, involved in heat stress responsiveness [64] were found in the promoter of *OsPIANK1*. GUS expression controlled by the full-length promoter was enhanced by inoculation of *M.oryzae*, a finding that confirmed our finding that overexpression of *OsPIANK1* enhanced the resistance of rice to *M.oryzae*. The region responsible for *M.oryzae* infection and response to exogenous SA was between −563 bp and −249 bp, where only one ERE and one TCA-element was found, suggesting that this TCA-element was responsible for the response of *OsPIANK1 to M.oryzae* infection and that this TCA-element mediated SA-signaling pathway is sufficient for the response of *OsPIANK1* to *M.oryzae* infection. This result is consistent with previous studies that SA is important in modulation of the redox balance and protection of rice plants from the oxidative stress caused by infection of *Magnaporthe grisea* [65] or *Xanthomonas oryzae* [66]. The regions responsible for the MeJA response in the promoter of *OsPIANK1* located between −1985 bp and −907 bp, where the CGTCA-box, JERE, the GCC-box, and MBS were located. Among these *cis*-elements, JERE and CGTCA-box *cis*-element may be responsible for the response of *OsPIANK1* to exogenous application of MeJA. In addition, MBS may play a role in the response of *OsPIANK1* to exogenous application of MeJA. In support of this, a myb gene that may bind to MBS was induced by jasmonic acid [67].

The data of the present study also suggested that the *Os*PIANK1 protein targeted to the nuclei and acted as a positive regulator in basal defense in rice. The latter conclusion was supported by the findings that inoculation of the WT, Nipponbare, with spores of *M.oryzae* strain guy11 caused a compatible reaction. Resistance to *M.oryzae* attack was greater in *OsPIANK*-OE rice plants than in WT, as manifested by fewer, smaller lesions, less *M. oryzae*, and greater vigor at 30 days after inoculation. These results indicated that *OsPIANK1* was a regulator of basal defense or PTI, although overexpression did not confer complete resistance.

The transcriptional expression of *NH1, WKRY13, PAL, AOS, PR1b,* and *PR5* were consistently enhanced by the overexpression of *OsPIANK1*. Of these genes, *NH1, WKRY13,* and *PR5* are either SA-dependent or involved in the regulation of SA [68]–[70], and *PAL* is associated with SA biosynthesis and induced by SA application [71]. *AOS2* is involved in JA synthesis [72]–[74] and *PR1b* has been reported to be associated with both JA and SA signaling pathways [69]. These results strongly suggest that *OsPIANK1* primes the expression of SA- and JA-responsive endogenous genes. Although the activated immune responses in ETI are more prolonged and robust than those in PTI [75], differences observed between compatible and incompatible interactions are temporal and quantitative, not qualitative [47], [48]. Common defense signaling molecules such as SA and JA are often common to both PTI and ETI [75], but the way they use the common network is very different: synergistic relationships among the signaling sectors are evident in PTI, compensatory relationships among the sectors are dominate in ETI [2]. Thus, the co-upregulation of JA- and SA-dependent genes by the overexpression of *OsPIANK1* is consistent with the role of *OsPIANK1* as a regulator in PTI or basal defense.

*OsPIANK1* was late up-regulated by infection of rice plants with the leaf blight pathogen (*Xoo*) and had different expression patterns against the attack of *Xoo* in the leaf-blight resistant cultivar IET8585 and the leaf-blight susceptible cultivar IR24 [44], as would be predicted. In the present study, *OsPIANK1* was cloned from Nipponbare, a japonica rice cultivar susceptible to *M.oryzae* and *Xoo.* Therefore, its expression patterns in response to pathogen attack and its function in disease resistance might be different from its analogues in resistant cultivars. Characterization of analogues of *OsPIANK1* should supply new insight into its function and underlying mechanism in rice immunity.

## References

[1] Hein I, Gilroy EM, Armstrong MR, Birch PR (2009) The zig-zag-zig in oomycete-plant interactions. Mol Plant Pathol 10: 547–562. 19523107 10.1111/j.1364-3703.2009.00547.x PMC6640229

[2] Tsuda K, Sato M, Stoddard T, Glazebrook J, Katagiri F (2009) Network properties of robust immunity in plants. PLoS Genet 5: e1000772. 20011122 10.1371/journal.pgen.1000772 PMC2782137

[3] Zhang J, Lu H, Li X, Li Y, Cui H, et al (2010) Effector-triggered and pathogen-associated molecular pattern-triggered immunity differentially contribute to basal resistance to *Pseudomonas syringae* . Mol Plant Microbe Interact 23: 940–948. 20521956 10.1094/MPMI-23-7-0940

[4] Thomma BP, Nurnberger T, Joosten MH (2011) Of PAMPs and effectors: the blurred PTI-ETI dichotomy. Plant Cell 23: 4–15. 21278123 10.1105/tpc.110.082602 PMC3051239

[5] Jacques A, Ghannam A, Erhardt M, de Ruffray P, Baillieul F, et al (2006) *NtLRP1*, a tobacco leucine-rich repeat gene with a possible role as a modulator of the hypersensitive response. Mol Plant Microbe Interact 19: 747–757. 16838787 10.1094/MPMI-19-0747

[6] Jung HW, Hwang BK (2007) The leucine-rich repeat (LRR) protein, CaLRR1, interacts with the hypersensitive induced reaction (HIR) protein, CaHIR1, and suppresses cell death induced by the CaHIR1 protein. Mol Plant Pathol 8: 503–514. 20507517 10.1111/j.1364-3703.2007.00410.x

[7] Sacco MA, Mansoor S, Moffett P (2007) A RanGAP protein physically interacts with the NB-LRR protein Rx, and is required for Rx-mediated viral resistance. Plant J 52: 82–93. 17655649 10.1111/j.1365-313X.2007.03213.x

[8] Yang Y, Zhang Y, Ding P, Johnson K, Li X (2012) The ankyrin-repeat transmembrane protein BDA1 functions downstream of the receptor-like protein SNC2 to regulate plant immunity. Plant Physiol 159: 1857–1865. 22740615 10.1104/pp.112.197152 PMC3425218

[9] Wu T, Tian Z, Liu J, Yao C, Xie C (2009) A novel ankyrin repeat-rich gene in potato, *Star*, involved in response to late blight. Biochem Genet 47: 439–450. 19396628 10.1007/s10528-009-9238-2

[10] Zhang H, Li X, Zhang Y, Kuppu S, Shen G (2010) Is AKR2A an essential molecular chaperone for a class of membrane-bound proteins in plants? Plant Signal Behav 5: 1520–1522. 21057222 10.4161/psb.5.11.13714 PMC3115272

[11] Shen G, Kuppu S, Venkataramani S, Wang J, Yan J, et al (2010) ANKYRIN REPEAT-CONTAINING PROTEIN 2A is an essential molecular chaperone for peroxisomal membrane-bound ASCORBATE PEROXIDASE3 in *Arabidopsis* . Plant Cell 22: 811–831. 20215589 10.1105/tpc.109.065979 PMC2861468

[12] Huang J, Zhao X, Yu H, Ouyang Y, Wang L, et al (2009) The ankyrin repeat gene family in rice: genome-wide identification, classification and expression profiling. Plant Mol Biol 71: 207–226. 19609685 10.1007/s11103-009-9518-6

[13] Becerra C, Jahrmann T, Puigdomenech P, Vicient CM (2004) Ankyrin repeat-containing proteins in *Arabidopsis*: characterization of a novel and abundant group of genes coding ankyrin-transmembrane proteins. Gene 340: 111–121. 15556299 10.1016/j.gene.2004.06.006

[14] Garcion C, Guilleminot J, Kroj T, Parcy F, Giraudat J, et al (2006) AKRP and EMB506 are two ankyrin repeat proteins essential for plastid differentiation and plant development in *Arabidopsis* . Plant J 48: 895–906. 17092312 10.1111/j.1365-313X.2006.02922.x

[15] Norberg M, Holmlund M, Nilsson O (2005) The *BLADE ON PETIOLE* genes act redundantly to control the growth and development of lateral organs. Development 132: 2203–2213. 15800002 10.1242/dev.01815

[16] Ha CM, Jun JH, Nam HG, Fletcher JC (2004) BLADE-ON-PETIOLE1 encodes a BTB/POZ domain protein required for leaf morphogenesis in *Arabidopsis thaliana* . Plant Cell Physiol 45: 1361–1370. 15564519 10.1093/pcp/pch201

[17] Zhang H, Scheirer DC, Fowle WH, Goodman HM (1992) Expression of antisense or sense RNA of an ankyrin repeat-containing gene blocks chloroplast differentiation in *Arabidopsis* . Plant Cell 4: 1575–1588. 1281700 10.1105/tpc.4.12.1575 PMC160243

[18] Bottner S, Iven T, Carsjens CS, Droge-Laser W (2009) Nuclear accumulation of the ankyrin repeat protein ANK1 enhances the auxin-mediated transcription accomplished by the bZIP transcription factors BZI-1 and BZI-2. Plant J 58: 914–926. 19220790 10.1111/j.1365-313X.2009.03829.x

[19] Stone SL, Williams LA, Farmer LM, Vierstra RD, Callis J (2006) KEEP ON GOING, a RING E3 ligase essential for *Arabidopsis* growth and development, is involved in abscisic acid signaling. Plant Cell 18: 3415–3428. 17194765 10.1105/tpc.106.046532 PMC1785414

[20] Huang J, Chen F, Del Casino C, Autino A, Shen M, et al (2006) An ankyrin repeat-containing protein, characterized as a ubiquitin ligase, is closely associated with membrane-enclosed organelles and required for pollen germination and pollen tube growth in lily. Plant Physiol 140: 1374–1383. 16461387 10.1104/pp.105.074922 PMC1435812

[21] Nodzon LA, Xu WH, Wang Y, Pi LY, Chakrabarty PK, et al (2004) The ubiquitin ligase XBAT32 regulates lateral root development in *Arabidopsis* . Plant J 40: 996–1006. 15584963 10.1111/j.1365-313X.2004.02266.x

[22] Kim DH, Xu ZY, Na YJ, Yoo YJ, Lee J, et al (2011) Small heat shock protein Hsp17.8 functions as an AKR2A cofactor in the targeting of chloroplast outer membrane proteins in *Arabidopsis* . Plant Physiol 157: 132–146. 21730198 10.1104/pp.111.178681 PMC3165864

[23] Bae W, Lee YJ, Kim DH, Lee J, Kim S, et al (2008) AKR2A-mediated import of chloroplast outer membrane proteins is essential for chloroplast biogenesis. Nat Cell Biol 10: 220–227. 18193034 10.1038/ncb1683

[24] Cui YL, Jia QS, Yin QQ, Lin GN, Kong MM, et al (2011) The GDC1 gene encodes a novel ankyrin domain-containing protein that is essential for grana formation in *Arabidopsis* . Plant Physiol 155: 130–141. 21098677 10.1104/pp.110.165589 PMC3075748

[25] Yoo J, Shin DH, Cho MH, Kim TL, Bhoo SH, et al (2011) An ankyrin repeat protein is involved in anthocyanin biosynthesis in *Arabidopsis* . Physiol Plant 142: 314–325. 21395597 10.1111/j.1399-3054.2011.01468.x

[26] Kumagai H, Hakoyama T, Umehara Y, Sato S, Kaneko T, et al (2007) A novel ankyrin-repeat membrane protein, IGN1, is required for persistence of nitrogen-fixing symbiosis in root nodules of *Lotus japonicus* . Plant Physiol 143: 1293–1305. 17277093 10.1104/pp.106.095356 PMC1820915

[27] Pumplin N, Mondo SJ, Topp S, Starker CG, Gantt JS, et al (2010) *Medicago truncatula* Vapyrin is a novel protein required for arbuscular mycorrhizal symbiosis. Plant J 61: 482–494. 19912567 10.1111/j.1365-313X.2009.04072.x

[28] Levy A, Guenoune-Gelbart D, Epel BL (2007) beta-1,3-Glucanases: Plasmodesmal Gate Keepers for Intercellular Communication. Plant Signal Behav 2: 404–407. 19704615 10.4161/psb.2.5.4334 PMC2634228

[29] Yu F, Shi J, Zhou J, Gu J, Chen Q, et al (2010) ANK6, a mitochondrial ankyrin repeat protein, is required for male-female gamete recognition in *Arabidopsis thaliana* . Proc Natl Acad Sci U S A 107: 22332–22337. 21123745 10.1073/pnas.1015911107 PMC3009778

[30] Gu Y, Innes RW (2011) The KEEP ON GOING protein of *Arabidopsis* recruits the ENHANCED DISEASE RESISTANCE1 protein to trans-Golgi network/early endosome vesicles. Plant Physiol 155: 1827–1838. 21343429 10.1104/pp.110.171785 PMC3091131

[31] Zhang X, Li D, Zhang H, Wang X, Zheng Z, et al (2010) Molecular characterization of rice OsBIANK1, encoding a plasma membrane-anchored ankyrin repeat protein, and its inducible expression in defense responses. Mol Biol Rep 37: 653–660. 19288292 10.1007/s11033-009-9507-5

[32] Xie C, Zhou X, Deng X, Guo Y (2010) PKS5, a SNF1-related kinase, interacts with and phosphorylates NPR1, and modulates expression of WRKY38 and WRKY62. J Genet Genomics 37: 359–369. 20621018 10.1016/S1673-8527(09)60054-0

[33] Wang YS, Pi LY, Chen X, Chakrabarty PK, Jiang J, et al (2006) Rice XA21 binding protein 3 is a ubiquitin ligase required for full *Xa21*-mediated disease resistance. Plant Cell 18: 3635–3646. 17172358 10.1105/tpc.106.046730 PMC1785399

[34] AbuQamar S, Chen X, Dhawan R, Bluhm B, Salmeron J, et al (2006) Expression profiling and mutant analysis reveals complex regulatory networks involved in *Arabidopsis* response to *Botrytis* infection. Plant J 48: 28–44. 16925600 10.1111/j.1365-313X.2006.02849.x

[35] Lu H, Rate DN, Song JT, Greenberg JT (2003) ACD6, a novel ankyrin protein, is a regulator and an effector of salicylic acid signaling in the *Arabidopsis* defense response. Plant Cell 15: 2408–2420. 14507999 10.1105/tpc.015412 PMC197305

[36] Fridborg I, Grainger J, Page A, Coleman M, Findlay K, et al (2003) TIP, a novel host factor linking callose degradation with the cell-to-cell movement of potato virus X. Mol Plant Microbe Interact. 16: 132–140. 10.1094/MPMI.2003.16.2.132 12575747

[37] Gao W, Li HY, Xiao S, Chye ML (2010) Protein interactors of acyl-CoA-binding protein ACBP2 mediate cadmium tolerance in *Arabidopsis* . Plant Signal Behav 5: 1025–1027. 20657176 10.4161/psb.5.8.12294 PMC3115187

[38] Seong ES, Cho HS, Choi D, Joung YH, Lim CK, et al (2007) Tomato plants overexpressing *CaKR1* enhanced tolerance to salt and oxidative stress. Biochem Biophys Res Commun 363: 983–988. 17927963 10.1016/j.bbrc.2007.09.104

[39] Dong X (2004) The role of membrane-bound ankyrin-repeat protein ACD6 in programmed cell death and plant defense. Sci STKE 2004: pe6. 14983101 10.1126/stke.2212004pe6

[40] Lee SC, Lan WZ, Kim BG, Li L, Cheong YH, et al (2007) A protein phosphorylation/dephosphorylation network regulates a plant potassium channel. Proc Natl Acad Sci U S A 104: 15959–15964. 17898163 10.1073/pnas.0707912104 PMC2000415

[41] Carvalho SD, Saraiva R, Maia TM, Abreu IA, Duque P (2012) XBAT35, a novel *Arabidopsis* RING E3 ligase exhibiting dual targeting of its splice isoforms, is involved in ethylene-mediated regulation of apical hook curvature. Mol Plant 5: 1295–1309. 22628544 10.1093/mp/sss048

[42] Li HY, Chye ML (2004) Arabidopsis Acyl-CoA-binding protein ACBP2 interacts with an ethylene-responsive element-binding protein, AtEBP, via its ankyrin repeats. Plant Mol Biol 54: 233–243. 15159625 10.1023/B:PLAN.0000028790.75090.ab

[43] Choi MS, Kim MC, Yoo JH, Moon BC, Koo SC, et al (2005) Isolation of a calmodulin-binding transcription factor from rice (*Oryza sativa* L.). J Biol Chem 280: 40820–40831. 16192280 10.1074/jbc.M504616200

[44] Kottapalli KR, Rakwal R, Satoh K, Shibato J, Kottapalli P, et al (2007) Transcriptional profiling of *indica* rice cultivar IET8585 (Ajaya) infected with bacterial leaf blight pathogen *Xanthomonas oryzae pv oryzae* . Plant Physiol Biochem 45: 834–850. 17870590 10.1016/j.plaphy.2007.07.013

[45] Katagiri F (2004) A global view of defense gene expression regulation–a highly interconnected signaling network. Curr Opin Plant Biol 7: 506–511. 15337092 10.1016/j.pbi.2004.07.013

[46] Eulgem T (2005) Regulation of the *Arabidopsis* defense transcriptome. Trends Plant Sci 10: 71–78. 15708344 10.1016/j.tplants.2004.12.006

[47] Maleck K, Levine A, Eulgem T, Morgan A, Schmid J, et al (2000) The transcriptome of *Arabidopsis thaliana* during systemic acquired resistance. Nat Genet 26: 403–410. 11101835 10.1038/82521

[48] Tao Y, Xie Z, Chen W, Glazebrook J, Chang HS, et al (2003) Quantitative nature of *Arabidopsis* responses during compatible and incompatible interactions with the bacterial pathogen *Pseudomonas syringae.* . Plant Cell 15: 317–330. 12566575 10.1105/tpc.007591 PMC141204

[49] Ramonell K, Berrocal-Lobo M, Koh S, Wan J, Edwards H, et al (2005) Loss-of-function mutations in chitin responsive genes show increased susceptibility to the powdery mildew pathogen *Erysiphe cichoracearum* . Plant Physiol 138: 1027–1036. 15923325 10.1104/pp.105.060947 PMC1150417

[50] Knoth C, Ringler J, Dangl JL, Eulgem T (2007) *Arabidopsis WRKY70* is required for full *RPP4*-mediated disease resistance and basal defense against *Hyaloperonospora parasitica* . Mol Plant Microbe Interact 20: 120–128. 17313163 10.1094/MPMI-20-2-0120

[51] Knoth C, Salus MS, Girke T, Eulgem T (2009) The synthetic elicitor 3,5-dichloroanthranilic acid induces *NPR1*-dependent and *NPR1*-independent mechanisms of disease resistance in *Arabidopsis* . Plant Physiol 150: 333–347. 19304930 10.1104/pp.108.133678 PMC2675713

[52] Curtis MD, Grossniklaus U (2003) A gateway cloning vector set for high-throughput functional analysis of genes in planta. Plant Physiol 133: 462–469. 14555774 10.1104/pp.103.027979 PMC523872

[53] Toki S, Hara N, Ono K, Onodera H, Tagiri A, et al (2006) Early infection of scutellum tissue with *Agrobacterium* allows high-speed transformation of rice. Plant J 47: 969–976. 16961734 10.1111/j.1365-313X.2006.02836.x

[54] Jefferson RA, Kavanagh TA, Bevan MW (1987) GUS fusions: beta-glucuronidase as a sensitive and versatile gene fusion marker in higher plants. EMBO J 6: 3901–3907. 3327686 10.1002/j.1460-2075.1987.tb02730.x PMC553867

[55] Livak KJ, Schmittgen TD (2001) Analysis of relative gene expression data using real-time quantitative PCR and the 2(-Delta Delta C(T)) Method. Methods 25: 402–408. 11846609 10.1006/meth.2001.1262

[56] Qi M, Yang Y (2002) Quantification of *Magnaporthe grisea* during infection of rice plants using real-time polymerase chain reaction and northern blot/phosphoimaging analyses. Phytopathology 92: 870–876. 18942966 10.1094/PHYTO.2002.92.8.870

[57] Veronese P, Nakagami H, Bluhm B, Abuqamar S, Chen X, et al (2006) The membrane-anchored *BOTRYTIS-INDUCED KINASE1* plays distinct roles in *Arabidopsis* resistance to necrotrophic and biotrophic pathogens. Plant Cell 18: 257–273. 16339855 10.1105/tpc.105.035576 PMC1323497

[58] Wang Q, Yuan F, Pan Q, Li M, Wang G, et al (2010) Isolation and functional analysis of the *Catharanthus roseus deacetylvindoline-4-O-acetyltransferase* gene promoter. Plant Cell Rep 29: 185–192. 20035334 10.1007/s00299-009-0811-2

[59] van der Fits L, Memelink J (2001) The jasmonate-inducible AP2/ERF-domain transcription factor *ORCA3* activates gene expression via interaction with a jasmonate-responsive promoter element. Plant J 25: 43–53. 11169181 10.1046/j.1365-313x.2001.00932.x

[60] Itzhaki H, Maxson JM, Woodson WR (1994) An ethylene-responsive enhancer element is involved in the senescence-related expression of the carnation glutathione-S-transferase (*GST1*) gene. Proc Natl Acad Sci U S A 91: 8925–8929. 8090746 10.1073/pnas.91.19.8925 PMC44719

[61] Ohme-Takagi M, Shinshi H (1995) Ethylene-inducible DNA binding proteins that interact with an ethylene-responsive element. Plant Cell 7: 173–182. 7756828 10.1105/tpc.7.2.173 PMC160773

[62] Salazar M, Gonzalez E, Casaretto JA, Casacuberta JM, Ruiz-Lara S (2007) The promoter of the *TLC1.1* retrotransposon from *Solanum chilense* is activated by multiple stress-related signaling molecules. Plant Cell Rep 26: 1861–1868. 17583815 10.1007/s00299-007-0375-y

[63] Li SF, Parish RW (1995) Isolation of two novel myb-like genes from *Arabidopsis* and studies on the DNA-binding properties of their products. Plant J 8: 963–972. 8580966 10.1046/j.1365-313x.1995.8060963.x

[64] Scharf KD, Rose S, Zott W, Schoffl F, Nover L (1990) Three tomato genes code for heat stress transcription factors with a region of remarkable homology to the DNA-binding domain of the yeast HSF. EMBO J 9: 4495–4501. 2148291 10.1002/j.1460-2075.1990.tb07900.x PMC552242

[65] Yang Y, Qi M, Mei C (2004) Endogenous salicylic acid protects rice plants from oxidative damage caused by aging as well as biotic and abiotic stress. Plant J 40: 909–919. 15584956 10.1111/j.1365-313X.2004.02267.x

[66] Yuan Y, Zhong S, Li Q, Zhu Z, Lou Y, et al (2007) Functional analysis of rice *NPR1*-like genes reveals that *OsNPR1/NH1* is the rice orthologue conferring disease resistance with enhanced herbivore susceptibility. Plant Biotechnol J 5: 313–324. 17309686 10.1111/j.1467-7652.2007.00243.x

[67] Lee MW, Qi M, Yang Y (2001) A novel jasmonic acid-inducible rice myb gene associates with fungal infection and host cell death. Mol Plant Microbe Interact 14: 527–535. 11310740 10.1094/MPMI.2001.14.4.527

[68] Chern M, Fitzgerald HA, Canlas PE, Navarre DA, Ronald PC (2005) Overexpression of a rice *NPR1* homolog leads to constitutive activation of defense response and hypersensitivity to light. Mol Plant Microbe Interact 18: 511–520. 15986920 10.1094/MPMI-18-0511

[69] Qiu D, Xiao J, Ding X, Xiong M, Cai M, et al (2007) *OsWRKY13* mediates rice disease resistance by regulating defense-related genes in salicylate- and jasmonate-dependent signaling. Mol Plant Microbe Interact 20: 492–499. 17506327 10.1094/MPMI-20-5-0492

[70] Ganesan V, Thomas G (2001) Salicylic acid response in rice: influence of salicylic acid on H_(2)_O_(2)_ accumulation and oxidative stress. Plant Sci 160: 1095–1106. 11337066 10.1016/s0168-9452(01)00327-2

[71] Blilou I, Ocampo JA, Garcia-Garrido JM (2000) Induction of *Ltp* (lipid transfer protein) and *Pal* (phenylalanine ammonia-lyase) gene expression in rice roots colonized by the arbuscular mycorrhizal fungus *Glomus mosseae* . J Exp Bot 51: 1969–1977. 11141171 10.1093/jexbot/51.353.1969

[72] Yoshii M, Yamazaki M, Rakwal R, Kishi-Kaboshi M, Miyao A, et al (2010) The NAC transcription factor RIM1 of rice is a new regulator of jasmonate signaling. Plant J 61: 804–815. 20015061 10.1111/j.1365-313X.2009.04107.x

[73] Glinwood R, Gradin T, Karpinska B, Ahmed E, Jonsson L, et al (2007) Aphid acceptance of barley exposed to volatile phytochemicals differs between plants exposed in daylight and darkness. Plant Signal Behav 2: 321–326. 19516995 10.4161/psb.2.5.4494 PMC2634203

[74] Maucher H, Hause B, Feussner I, Ziegler J, Wasternack C (2000) Allene oxide synthases of barley (*Hordeum vulgare* cv. Salome): tissue specific regulation in seedling development. Plant J 21: 199–213. 10743660 10.1046/j.1365-313x.2000.00669.x

[75] Tsuda K, Katagiri F (2010) Comparing signaling mechanisms engaged in pattern-triggered and effector-triggered immunity. Curr Opin Plant Biol 13: 459–465. 20471306 10.1016/j.pbi.2010.04.006

